# Evaluating the Safety of Early Return to Play After Metacarpal or Scaphoid Fracture: A Systematic Review

**DOI:** 10.7759/cureus.104379

**Published:** 2026-02-27

**Authors:** Calvin Wang, Jason Daniels, Daniel Devine, Michael Simon, David Kirschenbaum, Brian M Katt

**Affiliations:** 1 Department of Orthopaedic Surgery, Rutgers Robert Wood Johnson Medical School, New Brunswick, USA

**Keywords:** early return-to-play, fracture, metacarpal, scaphoid, union

## Abstract

This review summarizes reported outcomes and complications associated with early versus standard return-to-play timelines in athletic patients. Specifically, we focus on athletes with metacarpal and scaphoid fractures to determine whether significant differences in complications and outcomes exist depending on the time of return. Return-to-play timing varies within sports medicine, and a focused review addressing metacarpal and scaphoid fractures currently does not exist. Therefore, this review seeks to synthesize existing studies to highlight the implications of early return to play.

## Introduction and background

Timely return to play after athletic injury is of paramount importance. In professional athletes, metacarpal fractures account for 34.5% of reported specific hand and wrist injuries, while scaphoid fractures account for 7.1% [1]. Among National Collegiate Athletic Association (NCAA) athletes, metacarpal or phalangeal fractures represent 19.9% of hand and wrist injuries, and scaphoid fractures represent 4.1% [2]. Metacarpal fractures have the highest incidence in contact sports such as football, lacrosse, and hockey [3]. Similarly, scaphoid fractures have a high incidence among football players: approximately 1 out of 100 college football athletes will sustain a scaphoid fracture [4,5].

Traditional treatment protocols typically delay return to play, especially in contact sports, until union is observed on radiographs. However, a growing number of athletes are treated with "early" return-to-play protocols prior to fracture union. In this review, "early" return to play is defined as return prior to clinical or radiographic fracture union, whereas "traditional" return to play is defined as return after fracture union is achieved. The literature provides no clear guidance regarding the optimal timeline, as many variables, including the athlete's sport and position, fracture pattern, treatment method, and league regulations, have an effect.

Among those treated non-operatively, casts or orthoses are often employed upon return; however, physiologic demands on the wrist and hand vary across sports. Functional return with an upper extremity cast or orthosis may restrict sport-specific wrist motion, which can affect participation in sports such as basketball, hockey, or baseball, or in skilled football positions [5]. For metacarpal fractures, buddy taping, casts, padded splints, and gloves are commonly used for the healing bone [6].

Several cohort studies, chart reviews, and case series have been published documenting the return of athletes with metacarpal and scaphoid fractures. This systematic review aims to synthesize the current evidence regarding early return to play following metacarpal or scaphoid fractures, identify trends in reported outcomes and complication rates across treatment modalities, and highlight remaining gaps in the literature.

## Review

Methods

A systematic review was undertaken in accordance with the Preferred Reporting Items for Systematic Reviews and Meta-Analyses (PRISMA), and the protocol was registered in PROSPERO (ID: CRD42024624125) [7,8]. A comprehensive search was conducted in PubMed, Cochrane Libraries, and Embase on July 17, 2024, for articles with metacarpal and scaphoid fractures. Hand searching and citation chaining were not performed, which may have resulted in omission of relevant studies. However, the use of three major databases with broad search terms was intended to capture the majority of peer-reviewed literature addressing return-to-play outcomes in athletic metacarpal and scaphoid fractures. The utilized search string can be seen below:

("Return to Sport"[Mesh] OR "return to play"[tiab] OR "return to sport*"[tiab] OR "return to sporting activit*"[tiab] OR "sporting activity resumption"[tiab] OR "early return to sport*"[tiab]) AND (("Fractures"[Mesh] OR "Fracture Dislocation"[Mesh] OR “fracture*”[tiab]) AND ("Metacarpal"[Mesh] OR "metacarpal*"[tiab] OR "Hand Bones"[Mesh] OR "hand bone*"[tiab]) OR ("Scaphoid"[Mesh] OR “scaphoid*”[tiab] OR “Wrist Bones”[Mesh] OR “wrist bone*”[tiab]) OR ("Proximal Phalanges"[Mesh] OR "proximal phalange*"[tiab] OR “Finger Bones”[Mesh] OR “finger bone*”[tiab]))

In terms of inclusion, this study focused on selecting full-length, English text articles. Relevant article types included randomized controlled trials, cohort studies, and case series and case reports. Articles were required to include details of return-to-play timelines among athletes with acute fractures of the metacarpals and/or scaphoid.

With regard to exclusion, articles were removed if they were abstract-only or non-English. Studies reporting metacarpal or scaphoid fractures in non-athletes were also excluded, and fractures indirectly related to athletic trauma (e.g., stress fracture) were not analyzed in this study. Furthermore, studies documenting treatment of subacute or chronic fractures of the metacarpals or scaphoid were not considered. Finally, the review did not account for any surgical technique articles, review articles, cadaveric studies, nonhuman studies, systematic reviews, or meta-analyses.

**Figure 1:**
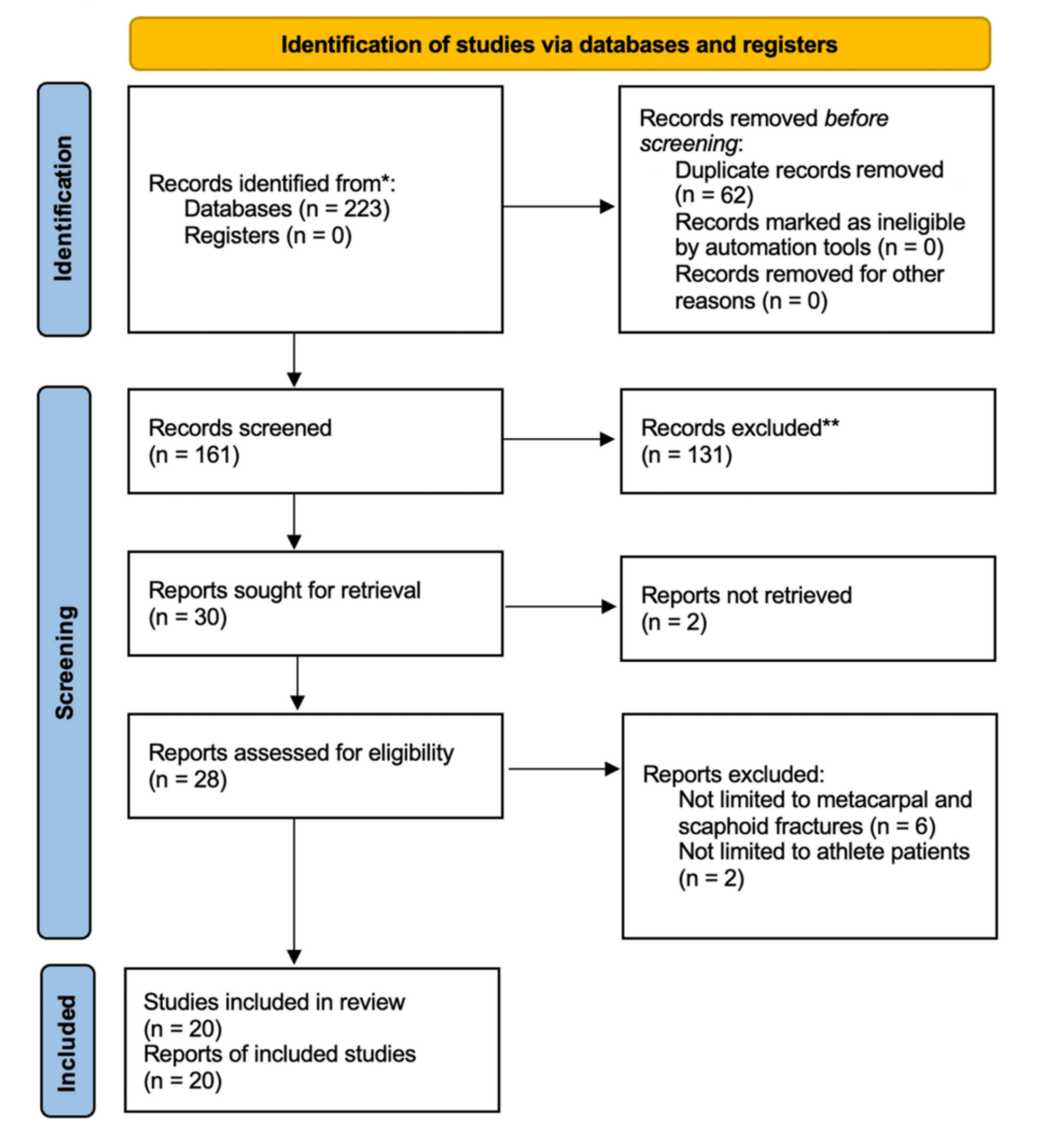
Flowchart of search and screening process.

The search results across all databases were exported into an EndNote library, where references were stored and organized [9]. After removing duplicates, the remaining studies were uploaded to Rayyan, a facilitative screening tool for systematic reviews [10]. Titles and abstracts were blindly screened by two reviewers for satisfactory fulfillment of inclusion and exclusion criteria, and any undecided or conflicted results were resolved by a third author. The full-text articles were then evaluated by two reviewers to determine the final papers for data extraction (Figure 1). 

Studies were characterized as early return to play if athletes returned prior to documented clinical or radiographic fracture union, whereas regular return to play was defined as return after union. This operational definition was used to facilitate cross-study comparison and does not imply biological equivalence across fracture types or treatment modalities. Because definitions of return to play and criteria for union were inconsistently reported across studies, categorization was based on timing relative to union when available or as described by the original authors. Return to play was also analyzed based on whether timing was reported relative to injury or surgery. For all included studies, two researchers evaluated study quality using the National Institutes of Health Quality Assessment Tool. Cohort studies were assessed by a 14-question NIH bias assessment, whereas case studies were assessed by a 9-question NIH bias assessment. Researchers consulted each other to align on final bias assignments for each article.

Quantitative synthesis was not performed due to substantial clinical and methodological heterogeneity across included studies. Sources of heterogeneity included variability in fracture location and pattern, operative versus non-operative management strategies, use and type of protective equipment, sport and positional demands, definitions of return to play, outcome measures, and follow-up duration. In addition, the predominance of retrospective case series and inconsistent reporting of outcomes limited the interpretability of pooled estimates or heterogeneity statistics such as *I*². For these reasons, quantitative pooling and formal heterogeneity assessment were not considered meaningful.

Results

The initial database search yielded 223 articles. Publications across all years were included to provide comprehensive analysis, and no outstanding studies were missed. After removal of duplicates, 161 studies underwent title and abstract screening. Following full-text review, 20 unique studies met inclusion criteria and were included in the final analysis. Two studies reported outcomes for both metacarpal and scaphoid fractures, resulting in 12 studies contributing to the metacarpal subgroup and 10 studies contributing to the scaphoid subgroup.

Across all included studies, data were extracted on fracture characteristics, management approach, sport, and player position, definition and timing of return to play, use of protective equipment, time to union when reported, and complications. Detailed study level summaries are provided in Appendix A.

The overall study quality was low to moderate. The majority of included studies were retrospective case series or retrospective cohort studies and commonly lacked control groups, predefined outcome measures, or standardized follow-up. Frequent sources of bias included selection bias related to athlete eligibility for early return to play, reporting bias due to inconsistent documentation of complications, and attrition bias from variable follow-up duration. Only a small number of studies met criteria for good methodological quality based on NIH quality assessment tools.

Metacarpal Fractures

A total of 471 athletic metacarpal fractures were identified across 12 studies (Table 1). Of these, 455 athletes returned to sport on an early return-to-play timeline prior to clinical or radiographic union, while 16 athletes returned after fracture union.

**Table 1:** Studies reporting return to play following metacarpal fracture in athletes. NCAA: National Collegiate Athletic Association.

Lead author (year)	No. of patients	Management	Sport	Average return-to-play time (weeks)	Return-to-play timeline	Complications	Study quality
Bougioukli et al., 2023 [11]	3	Operative	Football	0.9	Early	Not mentioned	Fair
Carender et al., 2019 [12]	15	Operative	All NCAA Sports	5.1	Early	Not mentioned	Fair
	79	Non-operative	All NCAA Sports	2.0	Early	Not mentioned	
Ellsasser et al., 1979 [13]	2	Operative	Football	1.0	Early	Not mentioned	Fair
Etier et al., 2015 [14]	20	Operative	Football	2.4	Early	Not mentioned	Fair
Kodama et al., 2014 [15]	8	Operative	3 Rugby 2 Soccer 1 Football 1 Handball 1 Judo	4.3	Early	Wound dehiscence (2)	Fair
Morse et al., 2017 [16]	13	Operative	Basketball	8.1	Regular	Not mentioned	Fair
	13	Non-operative	Basketball	3.7	Early	Not mentioned	
Parikh et al., 2022 [17]	5	Operative	Basketball	5.0	Early	Not mentioned	Good
Rettig et al., 1989 [18]	5	Operative	Football	1.9	Early	Not mentioned	Fair
	48	Non-operative	24 Football 14 Basketball 3 Baseball 7 Other	2.0	Early	Not mentioned	
Robertson et al., 2018 [19]	3	Operative	Soccer	18.3	Regular	Not mentioned	Good
	17	Non-operative	Soccer	4.6	Early	Not mentioned	
Sharareh et al., 2023 [20]	200	79 Operative 121 Non-operative	Football	2.1	Early	Not mentioned	Fair
Toronto et al., 1996 [21]	24	Non-operative	13 Football 3 Skiing 2 Baseball 2 Wrestling 4 Other	2.0	Early	Not mentioned	Fair
Yalizis et al., 2017 [22]	16	Operative	Australian Football	1.8	Early	Mild to moderate discomfort to rubbing (2)	Good

Across studies, early return to play following a metacarpal fracture typically occurred within two to three weeks of injury. The mean return-to-play time among early return athletes was 2.36 weeks, compared with 10.1 weeks among those returning after union. Early return was observed across a wide range of sports, including football, basketball, soccer, rugby, skiing, wrestling, and baseball.

Both operative and non-operative management strategies supported early return to play. Operatively treated athletes frequently returned within one to two weeks, particularly in contact sports where rigid fixation allowed early functional use with protective casting or padding. Non-operatively treated athletes also demonstrated early return, often around two weeks, typically with the use of protective splints, casts, or glove-based immobilization. In studies directly comparing treatment modalities, operative management tended to facilitate slightly earlier return, although substantial overlap existed between groups.

Sport type and positional demands influenced return timelines. Football players, particularly linemen and defensive players, consistently returned earlier than athletes in sports requiring greater fine motor control or wrist mobility, such as basketball or soccer. Several studies noted faster return among positions less dependent on grip strength or precise hand function [14,17,18]. In-season status also influenced return decisions, with in-season athletes more likely to return rapidly using protective equipment.

Across included metacarpal fracture studies, reported complications were uncommon and largely minor. No refractures, malunions, or nonunions were reported; however, follow-up duration and complication reporting varied across studies. Minor reported events included two cases of wound dehiscence and two cases of mild to moderate discomfort related to implant prominence [15,22]. Reported complications should not be interpreted as true incidence rates, as most included studies were retrospective case series without standardized follow-up or systematic complication reporting. These events are therefore best understood as reported adverse outcomes rather than reliable estimates of risk.

Scaphoid Fractures

A total of 208 athletic scaphoid fractures were identified across 10 studies (Table 2). Among these, 135 athletes returned to sport on an early return to play timeline, while 73 returned following fracture union.

**Table 2:** Description of studies analyzing return to play following scaphoid fracture in athletes.

Lead author (year)	No. of patients	Management	Sport	Average return-to-play time (weeks)	Return-to-play timeline	Complications	Study quality
Bougioukli et al., 2023 [11]	7	Operative	Not mentioned	9.6	Early	Not mentioned	Fair
Huene, 1979 [23]	4	Operative	Not mentioned	7.0	Early	Avascular necrosis (1)	Poor
McQueen et al., 2008 [24]	30	Operative	Not mentioned	6.4	Early	Not mentioned	Fair
	30	Non-operative	Not mentioned	15.5	Regular	Not mentioned	
Muramatsu et al., 2002 [25]	10	Operative	Soccer, Judo, Basketball, Baseball, Track, Boxing, Handball, Rugby, Badminton, Tennis	10.2	Early	Nonunion (1)	Fair
Rettig et al., 1994 [26]	18	Operative	Not mentioned	8.0	Early	Not mentioned	Good
	12	Non-operative	Not mentioned	4.3	Early	Nonunion (1)	
Rettig et al., 1996 [27]	12	Operative	8 Basketball 2 Baseball 2 Archery	5.8	Early	Nonunion (1)	Fair
Riester et al., 1985 [28]	14	Non-operative	12 Football 1 Soccer 1 Basketball	Immediate	Early	Nonunion (2) Refracture (1)	Fair
Robertson and Wood, 2015 [29]	1	Operative	Cycling	7.0	Early	Not mentioned	Fair
Robertson et al., 2018 [19]	2	Operative	Soccer	8.5	Early	Screw infection (1)	Good
	2	Non-operative, then operative after nonunion	Soccer	40.0	Regular	Nonunion prior to operative fixation (2)	
	2	Non-operative, then operative after nonunion	Soccer	Not Mentioned	Regular		
	12	Non-operative	Soccer	12.7	Regular	Delayed union (2)	
Surucu and Kehribar, 2022 [30]	25	Operative	Not mentioned	7.0	Early	Not mentioned	Fair
	27	Non-operative	Not mentioned	11.0	Regular	Nonunion (1)	

Early return to play after a scaphoid fracture occurred at a mean of 6.25 weeks. Among early return athletes, operatively treated patients returned at a mean of 7.26 weeks, whereas 26 non-operatively treated athletes who returned early did so at a mean of 1.98 weeks, most commonly using cast immobilization. Athletes who returned on a regular timeline were all managed non-operatively and returned at a mean of 13.97 weeks.

Compared to metacarpal fractures, scaphoid fractures demonstrated greater variability in return timelines and higher complication rates. Early return to play was more commonly achieved following operative fixation, which consistently resulted in a faster return compared with cast immobilization alone, even when radiographic union had not yet occurred.

Complications were reported in both the early and regular return groups, including nonunion, avascular necrosis, refracture, and infection. Follow-up duration and complication reporting were inconsistent across studies. As with metacarpal fractures, these reported events should be interpreted descriptively rather than as comparative incidence rates due to study design limitations and variable reporting.

Fracture location appeared to influence outcomes in scaphoid fractures. Proximal pole fractures were disproportionately represented among cases of nonunion, refracture, and avascular necrosis, particularly in studies permitting very early or immediate return to play with cast immobilization.

Overall, early return to play following metacarpal fractures was common, rapid, and associated with minimal complications across treatment modalities and sports. Early return following scaphoid fractures was feasible in selected cases but demonstrated greater variability and a higher complication burden, particularly among non-operatively treated athletes and those with proximal pole fracture patterns.

Discussion

This systematic review evaluated the early versus regular return to play following metacarpal and scaphoid fractures in athletes. The findings suggest that early return to play is frequently pursued, particularly for metacarpal fractures, and does not appear to be associated with a high burden of reported complications compared with return after fracture union, within the limitations of the available evidence. However, interpretation of these findings requires careful consideration of what "early" return to play represents biologically and clinically, as well as recognition of important limitations within the available literature.

Return to play following fracture fixation may be best conceptualized as occurring along a continuum rather than a binary decision. The earliest phase involves return based almost entirely on mechanical stability provided by fixation hardware. In this stage, biological healing has not meaningfully occurred, and the implant bears most of the load. This approach was most commonly described in operatively treated metacarpal fractures, particularly in contact sports, where rigid fixation combined with external protection allowed athletes to resume participation within one to two weeks of injury [11,13,14,20].

The second phase represents the early functional return in the setting of incomplete clinical healing. In this scenario, fracture inflammation has diminished, pain is controlled, and range of motion and strength are sufficient for sport-specific tasks, but fracture tenderness may persist and biological healing is still early. Athletes returning during this phase often rely on protective immobilization, such as casts, splints, or padded gloves, to limit stress across the fracture site while allowing controlled participation. Many non-operatively treated metacarpal fractures and select scaphoid fractures returning with cast immobilization fall into this category [16,19,24].

The third phase reflects clinical healing without radiographic union. At this stage, athletes are pain-free, demonstrate functional strength and motion, and tolerate sport-specific demands, yet radiographs continue to show incomplete fracture consolidation. Several studies in both metacarpal and scaphoid fractures reported safe return to play during this interval, particularly following surgical fixation [11,15,17,23-25]. These findings support the concept that clinical recovery may precede radiographic healing and that functional readiness may be a more relevant determinant of return to play in selected cases.

The final phase corresponds to the traditional return to play following radiographic union, where biological healing is complete and external protection is no longer required. This approach was more commonly observed in non-operatively treated scaphoid fractures and in off-season athletes where the urgency to return was lower [16,19,24,30]. However, radiographic assessment of scaphoid union was not standardized across included studies, and plain radiographs may have limited sensitivity for confirming union in some cases.

Importantly, these phases are not consistently or explicitly defined across studies, contributing to ambiguity in the literature. Terms such as "early return," "return before union," and "return with protection" are used variably and often without clear reference to biological healing status, clinical symptoms, or radiographic findings. This lack of standardized terminology complicates comparisons across studies and underscores the need for more precise definitions in future research.

Fracture type played a significant role in return-to-play outcomes. Metacarpal fractures demonstrated consistently low complication rates across treatment modalities and return timelines. The relative simplicity of fracture biology, favorable vascularity, and tolerance for protective immobilization likely contribute to the safety of early return in these injuries. In contrast, scaphoid fractures exhibited greater variability in return timelines and higher complication rates, particularly among non-operatively treated athletes and proximal pole fractures. The scaphoid’s retrograde blood supply and prolonged healing time appear to limit the margin for aggressive return strategies, especially when biological healing has not adequately progressed [23].

Comparisons between metacarpal and scaphoid fractures should be interpreted with caution. Scaphoid fractures possess distinct biological characteristics, including retrograde blood supply and higher inherent risk of nonunion, which likely contribute to higher reported complication rates independent of return-to-play timing.

Several limitations must be acknowledged. Although we searched three major databases, we did not perform supplementary strategies such as hand‑searching reference lists, forward citation tracking, or reviewing grey literature and conference proceedings. As a result, some relevant studies may not have been identified. The available evidence was largely composed of retrospective case series and small observational cohorts, including case reports, which introduce substantial selection, reporting, and publication bias. These study designs also do not allow reliable estimation of complication incidence or comparative safety. Follow-up duration and methods for capturing complications were inconsistently reported, likely leading to under recognition of delayed adverse outcomes such as delayed union, nonunion, or refracture. Definitions of return-to-play readiness and fracture union varied across studies and were sometimes incompletely described, creating potential misclassification and limiting meaningful comparison across studies. In addition, marked clinical and methodological heterogeneity, including differences in fracture patterns, treatment approaches, protective equipment, sport demands, and outcome definitions, prevented quantitative synthesis. Therefore, our findings should be interpreted as descriptive trends rather than pooled risk estimates. Finally, comparisons between metacarpal and scaphoid fractures are inherently confounded by biological differences, such as the scaphoid's vascular supply and higher baseline nonunion risk, as well as treatment-selection factors that may preferentially allow early return in lower-risk injuries.

Selection and reporting bias also warrant consideration. Athletes selected for early return are likely those with more favorable fracture patterns, better fixation constructs, or lower perceived risk, which may bias outcomes toward success. Additionally, adverse outcomes following early return may be underreported, as athletes experiencing complications or failures may be less likely to be written up or published. This form of publication and reporting bias may contribute to the low complication rates observed across studies and should temper interpretation of the findings.

Despite these limitations, this review highlights important trends in contemporary sports fracture management. Early return to play following metacarpal fracture appears common and safe when appropriately selected and protected. Early return following scaphoid fracture may be feasible in select cases, particularly after surgical fixation, but requires greater caution due to fracture biology and higher complication risk. Ultimately, return-to-play decisions should be individualized, accounting for fracture characteristics, treatment method, sport-specific demands, and the phase of healing rather than reliance on radiographic milestones alone.

## Conclusions

Early return to play is increasingly utilized in athletic populations, particularly for metacarpal injuries, where available evidence describes rapid return across both operative and non-operative management when appropriate protection and athlete selection are employed. In contrast, scaphoid fractures demonstrate greater heterogeneity in return timelines and a higher risk profile, especially among non-operatively treated athletes and proximal pole fractures, underscoring the importance of fracture biology in guiding return decisions. Within the limitations of predominantly low-level evidence, early return to play following metacarpal fracture is commonly reported and reported complications were uncommon in the included literature, but the true incidence is uncertain due to study design and reporting limitations. Early return following scaphoid fracture may be feasible in selected cases but carries greater uncertainty due to fracture biology and higher inherent risk.

Future prospective studies with standardized return-to-play criteria, consistent outcome reporting, and adequate follow-up are needed to better define optimal timelines and risk stratification. Until then, return-to-play decisions should be individualized, integrating fracture characteristics, treatment stability, sport-specific demands, and clinical recovery rather than relying on radiographic union alone.

## Appendices

Appendix

The risk-of-bias assessments for cohort studies are presented in Table 3.

**Table 3:** Quality of cohort studies as assessed with the NIH Quality Assessment Tool for observational cohort and cross-sectional studies. CD: cannot determine; N: no; NA: not applicable; NR: not reported; Y: yes. Questions: 1. Was the research question or objective in this paper clearly stated? 2. Was the study population clearly specified and defined? 3. Was the participation rate of eligible persons at least 50%? 4. Were all the participants selected or recruited from the same or similar populations (including the same time period)? Were inclusion and exclusion criteria for being in the study prespecified and applied uniformly to all participants? 5. Was a sample size justification, power description, or variance and effect estimates provided? 6. For the analyses in this paper, were the exposure(s) of interest measured prior to the outcome(s) being measured? 7. Was the time frame sufficient so that one could reasonably expect to see an association between exposure and outcome if it existed? 8. For exposures that can vary in amount or level, did the study examine different levels of the exposure as related to the outcome (e.g., categories of exposure, or exposure measured as continuous variable)? 9. Were the exposure measures (independent variables) clearly defined, valid, reliable, and implemented consistently across all study participants? 10. Was the exposure(s) assessed more than once over time? 11. Were the outcome measures (dependent variables) clearly defined, valid, reliable, and implemented consistently across all study participants? 12. Were the outcome assessors blinded to the exposure status of participants? 13. Was loss to follow-up after baseline 20% or less? 14. Were key potential confounding variables measured and adjusted statistically for their impact on the relationship between exposure(s) and outcome(s)?

Study (year)	Assessment questions														
	1	2	3	4	5	6	7	8	9	10	11	12	13	14	Overall
Bougioukli et al., 2023 [11]	Y	Y	NR	Y	N	NA	N	NA	NA	NA	Y	N	NA	Y	Fair
Carender et al., 2019 [12]	Y	Y	Y	Y	N	Y	Y	NA	Y	NA	Y	N	NA	N	Fair
Ellsasser et al., 1979 [13]	Y	Y	Y	Y	NA	Y	Y	Y	Y	NA	N	N	Y	N	Fair
Etier et al., 2015 [14]	Y	Y	NA	Y	N	NA	N	NA	NA	NA	Y	N	Y	N	Fair
Kodama et al., 2014 [15]	Y	Y	NA	Y	N	Y	Y	Y	Y	NA	Y	N	Y	N	Fair
Morse et al., 2017 [16]	Y	Y	Y	Y	N	Y	NA	NA	Y	NA	Y	N	NA	N	Fair
Rettig et al., 1989 [18]	Y	Y	NA	Y	N	Y	Y	Y	Y	N	Y	N	Y	N	Fair
Robertson et al., 2018 [19]	Y	Y	Y	Y	N	Y	Y	Y	Y	NA	Y	N	Y	Y	Good
Sharareh et al., 2023 [20]	Y	Y	Y	Y	N	Y	NA	Y	Y	N	Y	N	Y	N	Fair
Toronto et al., 1996 [21]	Y	Y	Y	Y	N	Y	N	N	Y	Y	Y	N	Y	N	Fair
Yalizis et al., 2017 [22]	Y	Y	Y	Y	N	Y	Y	Y	Y	Y	Y	N	Y	N	Good
McQueen et al., 2008 [24]	Y	Y	N	N	N	Y	Y	Y	NA	CD	Y	NR	Y	Y	Fair
Muramatsu et al., 2002 [25]	Y	Y	N	Y	N	Y	Y	Y	Y	N	Y	N	Y	NR	Fair
Rettig et al., 1994 [26]	Y	Y	Y	Y	N	Y	Y	N	Y	Y	Y	Y	Y	N	Good
Rettig et al., 1996 [27]	Y	Y	Y	Y	N	Y	Y	N	Y	N	Y	N	Y	NR	Fair
Riester et al., 1985 [28]	Y	Y	NA	Y	N	Y	Y	Y	Y	N	Y	N	Y	N	Fair
Surucu and Kehribar, [30]	Y	Y	NA	Y	N	Y	Y	N	Y	N	Y	N	Y	N	Fair

The risk-of-bias assessments for case reports and case series are presented in Table 4.

**Table 4:** Quality of case reports and case series, as assessed with the NIH Quality Assessment Tool for case series studies. CD: cannot determine; N: no; NA: not applicable; NR: not reported; Y: yes. Questions: 1. Was the study question or objective clearly stated? 2. Was the study population clearly and fully described, including a case definition? 3. Were the cases consecutive? 4. Were the subjects comparable? 5. Was the intervention clearly described? 6. Were the outcome measures clearly defined, valid, reliable, and implemented consistently across all study participants? 7. Was the length of follow-up adequate? 8. Were the statistical methods well-described? 9. Were the results well-described?

Study (year)	Assessment questions									
	1	2	3	4	5	6	7	8	9	Overall
Parikh et al., 2022 [17]	Y	Y	Y	Y	Y	Y	Y	N	Y	Good
Huene, 1979 [23]	NA	Y	CD	NA	Y	Y	CD	NA	Y	Poor
Robertson and Wood, 2015 [29]	NA	Y	NA	NA	Y	Y	Y	N	Y	Fair

The PICO framework and systematic review search strategy are presented in Table 5.

**Table 5:** PICO and search strategy information. PICO: Population, Intervention, Control, and Outcome.

Population	Athlete patients with acute metacarpal and/or scaphoid fracture
Intervention	Early return to play before fracture union
Control	Regular return to play after fracture union
Outcome	Long-term, post-treatment complications
Search Strategy	("Return to Sport"[Mesh] OR "return to play"[tiab] OR "return to sport*"[tiab] OR "return to sporting activit*"[tiab] OR "sporting activity resumption"[tiab] OR "early return to sport*"[tiab]) AND (("Fractures"[Mesh] OR "Fracture Dislocation"[Mesh] OR “fracture*”[tiab]) AND ("Metacarpal"[Mesh] OR "metacarpal*"[tiab] OR "Hand Bones"[Mesh] OR "hand bone*"[tiab]) OR ("Scaphoid"[Mesh] OR “scaphoid*”[tiab] OR “Wrist Bones”[Mesh] OR “wrist bone*”[tiab]) OR ("Proximal Phalanges"[Mesh] OR "proximal phalange*"[tiab] OR “Finger Bones”[Mesh] OR “finger bone*”[tiab]))
